# A Comparison of Exogenous Promoter Activity at the *ROSA26* Locus Using a PhiC31 Integrase Mediated Cassette Exchange Approach in Mouse ES Cells

**DOI:** 10.1371/journal.pone.0023376

**Published:** 2011-08-11

**Authors:** Chiann-mun Chen, Jon Krohn, Shoumo Bhattacharya, Benjamin Davies

**Affiliations:** 1 Wellcome Trust Centre for Human Genetics, University of Oxford, Oxford, United Kingdom; 2 Department of Cardiovascular Medicine, University of Oxford, Oxford, United Kingdom; University of Massachusetts Medical, United States of America

## Abstract

The activities of nine ubiquitous promoters (*ROSA26*, CAG, CMV, CMVd1, UbC, EF1α, PGK, chicken β-actin and MC1) have been quantified and compared in mouse embryonic stem cells. To avoid the high variation in transgene expression which results from uncontrolled copy number and chromosomal position effects when using random insertion based transgenic approaches, we have adopted a PhiC31 integrase mediated cassette exchange method for the efficient insertion of transgenes at single copy within a defined and well characterized chromosomal position, *ROSA26*. This has enabled the direct comparison of constructs from within the same genomic context and allows a systematic and quantitative assessment of the strengths of the promoters in comparison with the endogenous ROSA26 promoter. The behavior of these exogenous promoters, when integrated at *ROSA26* in both sense and antisense orientations, reveals a large variation in their levels of activity. In addition, a subset of promoters, EF1α, UbC and CAG, show an increased activity in the sense orientation as a consequence of integration. Transient transfection experiments confirmed these observations to reflect integration dependent effects and also revealed significant differences in the behaviour of these promoters when delivered transiently or stably. As well as providing an important reference which will facilitate the choice of an appropriate promoter to achieve the desired level of expression for a specific research question, this study also demonstrates the suitability of the cassette exchange methodology for the robust and reliable expression of multiple variant transgenes in ES cells.

## Introduction

Stem cells and in particular embryonic stem (ES) cells are frequently used in gene function studies, where transgenes are introduced under the control of exogenous promoters. The functional consequences of transgene expression can be assessed in stem cells directly, in their in vitro differentiated cell types or are frequently used for the generation of genetically modified mouse models. The level of transgene expression is determined by the promoter choice and selecting the correct promoter for a physiologically relevant expression level for the particular experimental question can be critical. Up to now, literature which aids this choice is limited and existing comparisons of promoter strength are frequently contradictory or use methodologies which may be inappropriate for comparative studies.

Gene targeting technologies in ES cells permit the insertion of transgenic constructs within defined genomic docking sites [1], [2]. This refinement limits the high variability of expression which is frequently seen in models made by conventional pronuclear microinjection, where constructs are integrated at random and at varying copy number into the genome [3]. Particularly when comparisons between variant constructs are required, the targeted insertion of transgenic cassettes provides the most appropriate methodology, as the functional effects of construct variation can be assayed within a standardized genomic insertion site.

The *ROSA26* locus (*Gt(ROSA)26Sor*) has become established as the preferred docking site for the ubiquitous expression of transgenes. The ubiquitous transcriptional activity of the locus suggests that the genomic region is not affected by chromatin configurations that could lead to transcriptional repression of inserted transgenes, and consequently, it has become a reliable insertion site for exogenous transgenes. Simulating the original retroviral promoter trap insertion [4], transgenes can be introduced into the first intron of the *ROSA26* forward transcript where the presence of a strong splice acceptor allows the transgene expression to be driven by the endogenous ubiquitous promoter. In addition, through the use of loxP flanked transcriptional terminators [5] or the adoption of loxP inversion strategies [6], transgene expression can be controlled, both spatially and temporally, by Cre recombinase activity. Standardized targeting vectors for *ROSA26* are now readily available, some of which have been adapted for facilitated cloning methodologies such as the Gateway system [7], [8].

Despite the relatively high homologous recombination frequency reported for *ROSA26* targeting in ES cells, for the rapid comparison of multiple transgenes, insertion using this method can be rate limiting, especially with increasing transgene size. Recombinase mediated cassette exchange (RMCE) methodologies provide a means of rapidly exchanging sequences lying between specific recombinase target sequences at high efficiency, directly in ES cells. RMCE approaches have been reported for the *ROSA26* locus and provide a faster and more efficient method of site specific insertion [9].

In addition to ubiquitous transgene expression via the endogenous promoter, transgenic constructs harbouring exogenous promoters have been positioned at the *ROSA26* locus. The site appears to be an appropriate docking site for the expression of tetracycline inducible constructs [10] and exogenous ubiquitous promoter driven constructs [8]. However, the transcriptional complexity of the *ROSA26* locus [11], evidence of orientation dependent effects [12] and a lack of systematic studies have limited the general application of this method with respect to exogenous promoters.

In this study, we have adapted a PhiC31 integrase mediated cassette exchange approach to achieve a systematic comparison of the strengths of common ubiquitous promoters positioned within the *ROSA26* locus. Transcriptional interference from upstream promoter sequences has been limited by the use of an insulator element and orientation dependent effects have been analysed. This study will aid the selection of the appropriate strength of promoter for stem cell expression and highlights a general tool for the comparison of variant transgenes.

## Materials and Methods

### Plasmid construction

The promoter sequences for chicken β-actin [13], human polypeptide chain elongation factor 1α (EF1α) [14], mouse phosphoglycerate kinase (PGK) [15], human ubiquitin C (UbC) [16], polyoma enhancer/herpes simplex virus thymidine kinase (MC1) [17], immediate early enhancer of human cytomegalovirus (CMV), a deletion derivative of CMV (CMVd1) [18] and CMV immediate early enhancer/chicken β-actin promoter/rabbit β-globin intron composite promoter (CAG) [19] were generated by PCR, sequence verified, and cloned upstream of the firefly (*Photinus pyralis*) luciferase (Fluc) coding sequence into pcDNA3 (Invitrogen) which had been modified to remove the CMV promoter. The majority of the constructs were validated for activity by assaying for Fluc activity in transiently transfected HEK293T (ATCC CRL-11268), Cos7 (ATCC CRL-1651), C2C12 (ATCC CRL-1772) cells (Figure S1) and IDG26.10-3 ES [9] cells which confirmed the different strengths of the promoters. Co-transfection with a CMV promoter driven sea pansy (*Renilla reniformis*) luciferase (Rluc) was used to normalize for transfection efficiency and cell number.

A generic PhiC31 integrase mediated exchange vector, pCB92, was assembled by modifying pExchange4-CB9 (derived from pRMCE [9]) with the insertion of a murine H19 insulator [20], obtained from pWHERE (Invivogen), together with a synthesized polylinker of unique sites to facilitate cloning. The exchange constructs were subsequently generated by subcloning the various constructs consisting of promoter, Fluc coding sequence and bovine growth hormone polyadenylation sequence, from the modified pcDNA3 plasmids (above) into pCB92 (PCR primers and cloning details available upon request).

To generate ES cells in which firefly luciferase is expressed under the control of the endogenous *ROSA26* promoter, a further PhiC31 integrase mediated exchange vector, pCB9-CB-Fluc, was generated by cloning the Fluc coding sequence downstream of the FRT site within pExchange4-CB9.

### Cell Culture

HEK293T, C2C12 and Cos7 cells were cultured in Dulbecco's modified Eagle's medium (DMEM; Sigma) supplemented with 10% fetal calf serum (Sigma), 1% penicillin/streptomycin (Invitrogen), and 4 mM L-Glutamine (Invitrogen). Embryonic stem cells were cultured in Knockout DMEM (Invitrogen) supplemented with 2 mM L-Glutamine (PAA), 1× non-essential amino acids (PAA), 0.1 mM β-mercaptoethanol (Sigma), 1000 U/ml ESGRO (Millipore) and 10% fetal bovine serum (Invitrogen). All cells were cultured at 37°C in a humidified atmosphere containing 5% CO_2_.

### PhiC31 integrase mediated cassette exchange at the ROSA26 locus

1×10^6^ IDG26.10-3 ES cells [9] were co-electroporated with 5 µg of exchange plasmid and 5 µg of pPhiC31o [21] using the Neon transfection system (Invitrogen) (3×1400 V, 10 ms) and plated on G418 resistant fibroblast feeder layers. After approximately 7 days of selection in 350 µg/ml G418, 16 resistant colonies were isolated per construct, expanded and screened for the correct cassette exchange event at the 5′ and 3′ ends using specific primers (5′ screen: 5′-CACGCTTCAAAAGCGCACGTCTG-3′ and 5′- GTTGTGCCCAGTCATAGCCGAATAG-3′ which yields a 280 bp product; 3′ screen: 5′- GCACTAGTTCTAGAGCGATCCCC-3′ and 5′- CGGGAGAAATGGATATGAAGTACTGGGC-3′ which yields a 518 bp product). The correct insertion and the presence of a single copy of the transgenic construct was confirmed for all clones used for assays in this study by Southern blotting of NheI digested genomic DNA and hybridization with a probe specific for the first 587 bps of firefly luciferase (data not shown).

### Generation of ROSA26 driven firefly luciferase ES cell clones

1×10^6^ IDG26.10-3 ES cells [9] were co-electroporated with 5 µg of pCB9-CB-Fluc and 5 µg of pPhiC31o [21], selected and screened as described above, generating ES cells in which the expression of Fluc by the endogenous *ROSA26* promoter is interrupted by an FRT flanked Neomycin selection cassette (*ROSA26*>Neo>Fluc).

2 independent *ROSA26*>Neo>Fluc ES cell clones were electroporated with 5 ug of pFLPo plasmid [21] using the Neon transfection system as described above and plated at low density on feeder layers. 6 days after electroporation, approximately 30 colonies were picked from each clone and screened for the deletion of the selection cassette by PCR. The resulting ES cells (*ROSA26*>Fluc) express Fluc under the control of the endogenous *ROSA26* promoter.

### Luciferase assay of stably transfected ES cell clones

Approximately 1×10^5^ cells per clone were seeded on 4 gelatine coated wells of a 96 well plate and assayed the following day. Cells were washed in PBS and lysed in lysis buffer containing 25 mM Tris Phosphate Buffer pH 7.8, 2 mM CDTA, 10% glycerol and 1% Triton X-100. 10 µl of lysate per sample were assayed in 100 µl of luciferase assay buffer containing 10 µg Potassium Luciferin, 15 mM MgSO_4_, 15 mM Potassium Phosphate Buffer pH 7.8, 4 mM EGTA pH 7.8 and supplemented with 20 µM ATP and 2 µM DTT. Luminescence was detected using a POLARstar Omega luminometer (BMG). 25 µl of lysate per sample were used to assay for total protein concentrations using the Bicinchoninic acid (BCA) protein assay kit (Pierce), as per manufacturer's instructions. Total protein concentrations were used to normalize the luminescence readings. Experiments were repeated at least three times on separate days.

### Firefly and Renillla Luciferase assay of transiently transfected cells

Approximately 1×10^5^ IDG26.10-3 ES cells, 2×10^4^ HEK293T, 1×10^4^ Cos7 and 1×10^4^ C2C12 cells were plated onto 96 well plates and transfected with 90 ng of exchange plasmid, 10 ng of a CMV driven *Renilla* luciferase plasmid and 0.2 µl of Fugene HD (Promega) per well for a total of 4 wells per construct. Firefly and *Renilla* luciferase readings were performed the following day. Briefly, cells were washed in PBS and lysed as described above. 10 µl of lysate per sample were used to sequentially assay for both, firefly and *Renilla* luciferase activities. Firefly luciferase was assayed in 100 µl of luciferase assay buffer as described above. Quantification of the firefly luminescence was followed by the *Renilla* luciferase reaction achieved by injection of 100 µl of PBS containing 10 µM of benzyl coelenterazine and 25 mM of Luciferase Inhibitor I (Merk Chemicals) used to quench the firefly luminescence. Fluc and Rluc activities were measured as relative light units with a POLARstar Omega luminometer (BMG). All values of firefly luciferase activity were normalized by using the *Renilla* luciferase activity for reference. All transfections and assays were repeated at least three times on separate days.

### Statistical Analysis

For each promoter tested, the lme4 package [22] for R (R Development Core Team 2004) was used to generate a hierarchical linear model for the data, predicting luminescence using experimental session, ES cell clone identity and orientation of the promoter-luciferase construct within the *ROSA26* locus as random effects variables. Using this analysis, the construct orientation parameter contributing to the data variation was estimated using a restricted likelihood ratio test (RLRsim package [23]). The Dunn-Šidák correction [24] was used to adjust the significance threshold (p<0.05) to compensate for multiple comparisons and consequently orientation was considered significant, on an experiment-wide basis, if the probability of an orientation dependent effect fell below 0.006.

## Results

A firefly luciferase (Fluc) coding sequence was cloned downstream of eight commonly used ubiquitous promoters; three viral promoters: CMV, CMVd1 and MC1; four vertebrate promoters: mouse PGK, chicken β-actin, human EF1α and human UbC; and the artificial compound CAG promoter. A ninth control construct which consisted of only the Fluc coding sequence without a promoter was also completed. These constructs were positioned in a sense and antisense orientation, downstream of a mouse H19 insulator element and were verified for expression by assaying for Fluc activity of transiently transfected HEK293T cells (Figure S2). The H19 insulator element was used at the 5′ of the inserted transgene to prevent transcriptional read-through and enhancer/silencer signals emanating from either the endogenous *ROSA26* promoter or the selection cassette (PGK-Neo-pA) from influencing the activity of the reporter cassettes. The *ROSA26* locus is also associated with an antisense transcript (known as transcript AS [11] or *Thumpd3*), but this transcript terminates approximately 8 kb downstream of the *ROSA26* integration site and, consequently, read-through transcription from the 3′ end of the construct was not expected.

**Figure 1:**
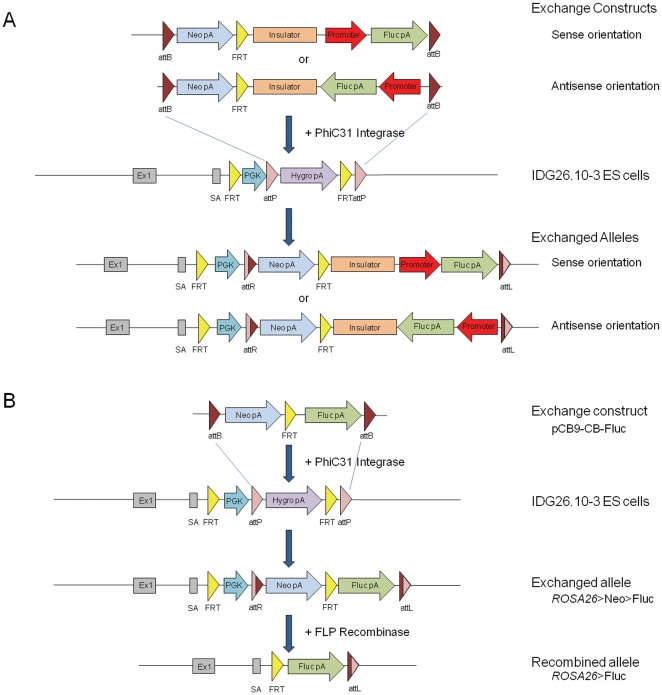
Vector configuration, *ROSA26* exchange and recombination events. (A) Exchange constructs harbouring the promoter-luciferase cassettes in either sense or antisense orientation were targeted to the *ROSA26* locus via PhiC31 integrase mediated cassette exchange. (B) A Fluc coding sequence was targeted to the *ROSA26* locus via PhiC31 integrase mediated cassette exchange. Flp recombinase mediated deletion of the selection cassette, brought the luciferase cassette into the context of the *ROSA26* forward transcript via the 5′ Splice Acceptor (SA).

The constructs in their respective orientations were targeted to the *ROSA26* locus via PhiC31 integrase mediated cassette exchange (Figure 1A), and 16 independent G418 resistant clones were isolated for each of these 18 different constructs. Multiple independent ES cell clones for each construct, integrated correctly at both the 5′ and 3′ ends, were identified and integration of a single copy of the transgenic construct was verified by Southern blot analysis (data not shown). The integration of transgenes of varying size occurred at high efficiency: 63.44% of the isolated G418 resistant colonies had integrated the transgenic construct appropriately at both ends, with the remaining 36.56% having only integrated the transgenic construct via a single 5′ attP×attB integration event, thus representing an insertion event rather than a true cassette exchange. Although these latter clones harbour the promoter reporter cassette at the *ROSA26* locus, an insertion event (rather than an exchange event) also leads to the insertion of the complete plasmid backbone sequence and subsequently were not used for further analysis. None of the clones identified had integrated the construct at random, suggesting the selection procedures adopted in the RMCE methodology to be highly reliable.

To provide a comparison for the quantification of expression in stable lines, ES cell clones harbouring the Fluc coding sequence under the control of the endogenous *ROSA26* promoter (*ROSA26*>Fluc) were engineered by Flp recombinase mediated deletion of the selection cassette in *ROSA26*>Neo>Fluc ES cells. This led to the Fluc coding sequence being brought into the context of the ubiquitously expressed *ROSA26* forward transcript via a splice acceptor site lying upstream of the deleted selection cassette (Figure 1B). Clones prior to Flp recombinase deletion (*ROSA26*>Neo>Fluc) were also used in the analysis as a further control.

**Figure 2:**
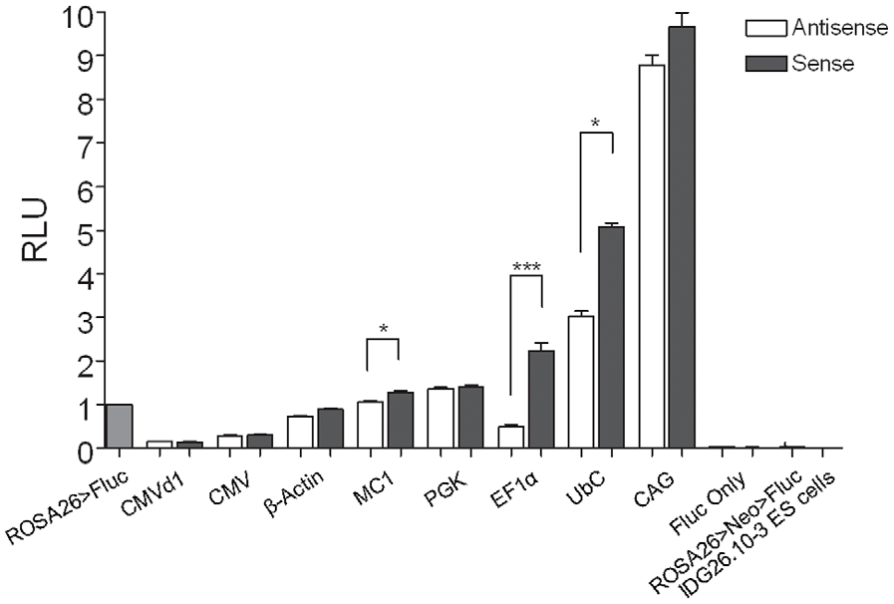
Relative promoter activities of Fluc reporter constructs stably integrated at *ROSA26*. Fluc expression cassettes under the control of different promoters were inserted into the *ROSA26* locus in both sense and antisense orientation by PhiC31 integrase mediated cassette exchange. Fluc activity from the exogenous promoters is expressed relative to that of luciferase under the control of the endogenous *ROSA26* promoter (*ROSA26*>Fluc). “Fluc Only” represents ES cells harbouring the promoterless construct, *ROSA26*>Neo>Fluc represents ES cells harbouring inactive *ROSA26* driven Fluc, prior to Flp deletion, and IDG26.10-3 ES cells are the “wild-type” ES cells used prior to the transgene insertion. Using multilevel modelling, the difference in activities between promoter-luciferase cassettes positioned in the sense and antisense orientations were found to be highly significant for EF1α (***p = 0.0007) and significant for UbC and MC1(*). Fluc assays were normalized to total protein concentrations and error bars represent the standard error of the mean from at least 3 independent experiments.

Fluc activity was quantified for multiple independent ES cell clones harbouring each of the 9 constructs in both orientiations and normalized against the activity of the control clones where the Fluc cassette was driven by the endogenous *ROSA26* promoter. Figure 2 summarizes the relative activity of each of the promoters in both sense and antisense orientations, in comparison to the endogenous *ROSA26* promoter. The control ES cell clones containing the promoterless luciferase cassettes in both orientations revealed only background levels of expression, providing no evidence for transcriptional read-through in either sense or antisense direction. In addition, expression from the endogenous *ROSA26* promoter was also found to be at background levels in the absence of Flp mediated deletion of the selection cassette.

Interestingly, in the absence of any apparent read-through transcription at the locus, some but not all of the promoters revealed significant orientation dependent differences in expression level. EF1α showed the strongest orientation dependent effects (p = 0.0007) with the sense orientation having approximately 4× the activity of the same cassette positioned in the antisense orientation. Similarly, UbC and MC1 driven expression also showed significantly higher expression when positioned in the sense orientation, although the effect was barely significant (p = 0.006 and p = 0.007, respectively) relative to the 0.006 p-value threshold obtained after adjusting for multiple comparisons.

**Figure 3:**
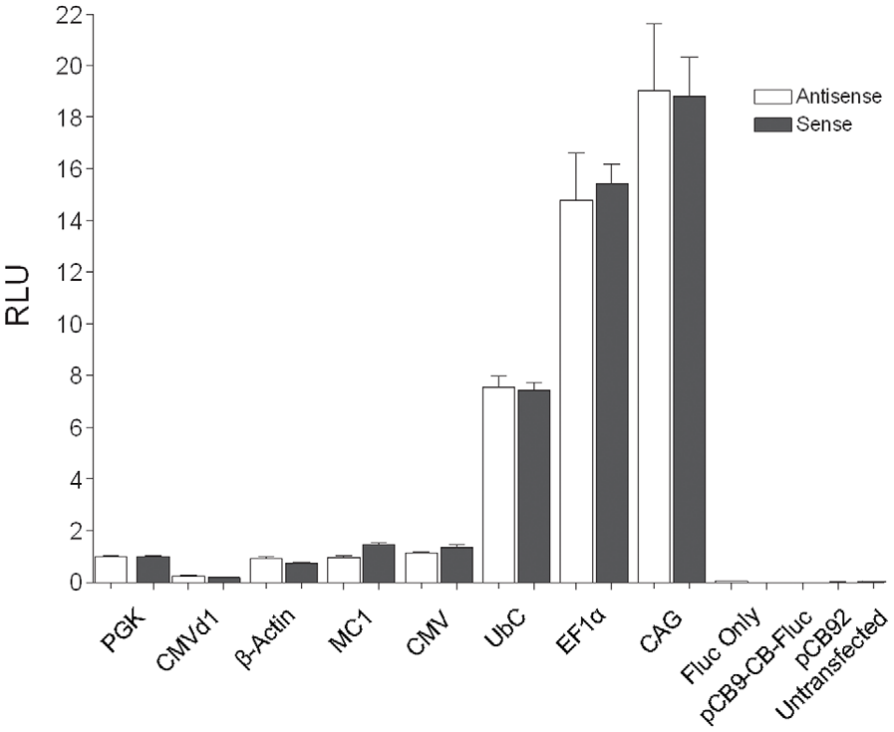
Relative promoter activities of transiently transfected Fluc reporter constructs. Fluc expression cassettes under the control of different promoters were transiently transfected into IDG26.10-3 ES cells, co-transfected with an Rluc expression plasmid to control for transfection efficiency and cell number. All Fluc readings were normalized against Rluc readings and promoter activities were expressed relative to the activity from the PGK promoter in the sense orientation for comparison. pCB9-CB-Fluc is the construct used to generate the cell line which expresses Fluc under the control of the endogenous *ROSA26* promoter. pCB92 is the empty exchange vector without a promoter or Fluc coding sequence. Error bars represent the standard error of the mean from at least 3 independent experiments.

To establish whether these orientation dependent effects were a result of chromosomal integration, the panel of exchange plasmids containing the various promoters driving Fluc was analysed in transient transfection assays in ES cells. Firefly and *Renilla* luciferase assays were performed by co-transfection of the Fluc reporter constructs with an Rluc expression plasmid used as a reference to normalize variations in transfection efficiency and cell number. No orientation dependent effects were observed with any of the promoter constructs tested, confirming that the previously observed differences are a consequence of stable integration at *ROSA26* rather than inherent properties of the expression vectors (Figure 3).

Interestingly, the behaviour of some of the promoters when delivered by transient transfection was found to differ from the activities observed when integrated at single copy into the genome. To allow a clearer comparison of the behaviour of the exogenous promoters in the different experimental contexts, the relative strengths of the promoter constructs introduced transiently or stably integrated into ES cells were ascertained and the results summarized in Table 1. In contrast to the previous results, and for ease of comparison, these results are tabulated using the PGK promoter in the sense orientation as a reference since the *ROSA26* promoter could only be used in the stable single copy integration analysis. The activity of the EF1α promoter revealed a considerably higher expression level when introduced transiently into the ES cells, with approximately 10 or 40 fold difference in activity when compared to the stably integrated constructs in the sense and antisense orientation respectively. Similarly, the activity of the CMV promoter was found to be much higher when transiently transfected than stably integrated into ES cells. These observations highlight the consequences that genomic integration can have on transgene expression for a number of commonly used ubiquitous promoters.

**Table 1:** Relative strengths of the different promoters in mouse ES cells, either stably integrated into *ROSA26* or transiently transfected.

	Stable	Transient
CMVd1 REV	0.1025	0.2454
CMVd1 FWD	0.0969	0.1825
CMV REV	0.2019	1.1434
CMV FWD	0.2161	1.3589
β-ACT REV	0.5137	0.9297
β-ACT FWD	0.6324	0.7448
*ROSA26*>Fluc	0.7125	n/a
MC1 REV	0.7493	0.9642
MC1 FWD	0.9038	1.4602
PGK REV	0.9709	0.9833
PGK FWD	1.0000	1.0000
EF1α REV	0.3455	14.7895
EF1α FWD	1.5941	15.4239
UBC REV	2.1604	7.5508
UBC FWD	3.6286	7.4364
CAG REV	6.2512	19.0293
CAG FWD	6.8933	18.8158
FLUC ONLY REV	0.0153	0.0448
FLUC ONLY FWD	0.0073	0.0030
*ROSA26*>Neo>Fluc	0.0125	n/a
pCB9-CB-Fluc Vector	n/a	0.0058
pCB92 Vector	n/a	0.0072

For comparison, all activities are calculated relative to those obtained from the PGK promoter in the sense orientation.

## Discussion

The strengths of 8 exogenous promoter constructs have been integrated at single copy in both forward and reverse orientation at the *ROSA26* locus using a PhiC31 integrase mediated cassette exchange approach. This has allowed the activity of these promoters to be quantified from within a consistent genomic integration site. The methodology used provides the most systematic and reliable method of comparing promoter activities and overcomes many of the shortcomings of previously published studies which have examined promoter strengths by using random integration approaches to generate stable cells lines [25]. In such studies, cell clones harbouring differing constructs integrated at different positions within the genome are directly compared. Any recorded differences may simply reflect differences in how permissive a particular chromosomal integration site is for transgene expression, rather than reflecting real differences in promoter strength.

The compound promoter, CAG, was found to yield the highest levels of expression at approximately 9–10 fold the level of the endogenous *ROSA26* promoter. These results are consistent with a previous report which quantified the *ROSA26* integrated CAG promoter activity as being approximately 8–10 fold the level of *ROSA26* [8].

The lowest promoter activities were observed for CMV and its deletion derivative, CMVd1, with activities approximating to 10–30% of the endogenous *ROSA26* promoter strength. The expression characteristics of CMV driven transgenes in stem cells have been the subject of some controversy. By transient transfection, an initial report suggested a complete absence of expression in ES cells with some degree of expression reappearing during in vitro differentiation [26], whereas subsequent reports confirmed expression both in transient [27], stably transfected [28] and adenoviral infected [29] clones, albeit at a relatively low level and with some degree of heterogeneity. The heterogeneity or mosaicism of stem cell CMV expression due to epigenetic silencing of randomly integrated transgenes is now well established [30] and may explain some of the inconsistencies seen in the published studies.

Of the vertebrate promoters tested, EF1α yielded robust expression levels approximately 4.5 fold higher than the *ROSA26* promoter when positioned in the sense orientation. Previous reports have also confirmed the moderate expression levels of EF1α in transient [26], stably transfected [28] and adenoviral infected [29] ES cell clones. In contrast to our results, a further study using lentiviral infection of mouse ES cells has suggested EF1α promoter strength to be approximately two fold higher than that of the CAG promoter [31]. However, although the authors controlled for copy number integration by quantitative PCR, the multiplicity of random integration events which occurs during lentiviral infection could still be a source of variation in the experimental read-out. UbC promoter driven expression was found to be 3–5 fold higher than the endogenous *ROSA26* promoter. Despite the well characterised ubiquitous expression observed in UbC driven transgenic mice [16], there is little data assessing the expression level of UbC in mouse ES cells. Strong transgene expression driven from UbC has however been confirmed in human stem cells [32] and human hematopoietic and murine mesenchymal progenitor cells [33].

PGK, chicken β-actin and MC1 promoter activity was found to be at a comparable level with that of the endogenous *ROSA26* promoter. There is little previous evidence directly comparing the activities of these promoters, although one study has assessed the strength of these promoters for achieving Cre recombinase mediated cassette deletions. This indirect evidence of promoter strength concluded that PGK and MC1 promoter activity are significantly lower than that of CAG and EF1α, in agreement with this study [34].

In terms of orientation dependent effects, two published studies have revealed that certain promoters behave differently if positioned in an antisense or sense orientation at the *ROSA26* locus. CAG promoter driven transgene expression was found to be at least ten fold higher in the antisense orientation than in the sense orientation [8] and CMV driven expression of the reverse tetracycline transactivator (rtTA) was inferred to be more robustly expressed in the antisense orientation [12]. Transcriptional read-through interference from the *ROSA26* sense transcripts was considered to be responsible for the lower level of expression of transgenes positioned in the sense orientation and there is some indirect evidence to suggest that this might indeed be the case. The deletion of a selection cassette lying between the endogenous *ROSA26* promoter and a CMV driven rtTA expression cassette reduced the activity of the transgene [12], suggesting that this selection cassette was previously screening the transgene from these interference effects to a certain extent.

Interestingly, the significant orientation dependent effects seen in this study suggest a trend towards increased expression in the sense orientation. However, the construct arrangement in this study includes the positioning of a mouse H19 insulator element between the expression cassette and the endogenous *ROSA26* promoter and, subsequently, it is likely that this serves to block all transcriptional read-through from the *ROSA26* promoter which may otherwise contribute to transcriptional interference with exogenous promoters positioned in the sense orientation. Indeed, the insertion of the promoterless luciferase cassettes in the sense orientation led to no discernible expression. Read-through transcription in the antisense orientation is not considered to influence the expression of transgenes inserted using his system as the recognised antisense transcript terminates 8 kb downstream of the transgene insertion site. In agreement, ES cell clones in which a promoterless luciferase cassette was positioned in the antisense orientation revealed only background levels of activity.

Despite the apparent lack of transcriptional read-through activity from the *ROSA26* promoter into the inserted transgenes in this system, significant orientation dependent effects were observed for the EF1α promoter with borderline significant effects being recorded for the MC1 and the UbC promoters. The results of the transient transfection assay, however, revealed no orientation dependent effects for these promoters, suggesting that these differences were not a consequence of transgene arrangement with respect to the plasmid backbone, the selection cassette, the insulator or the integration machinery. Instead, these effects must be dependent upon integration within the *ROSA26* locus and thus potentially reflect the accessibility of these promoter elements for transcription factors or steric considerations.

In conclusion, the results presented in this study quantify the strengths of commonly used ubiquitous promoters when integrated at single copy within the *ROSA26* locus. The study also serves to validate the PhiC31 integrase mediated cassette exchange approach for the analysis of multiple variant constructs and demonstrates the relative ease and robustness of performing multiple comparative analyses. It is becoming clear that conclusions drawn from studies using conventional, random-integration based methodologies are weakened by the position effects resulting from the varying integration site and lack of strict copy number control. The approach outlined in this study provides a powerful alternative for comparative transgenic analysis where the functional consequences of transgene variation can be assessed within a stable chromosomal context. The stem cells generated within the study can, in the future, be used for the generation of mouse models to assess the comparative strengths of these promoters in vivo and to determine whether the various promoters are equally active in all cell types.

## Supporting Information

Figure S1 Comparison of the strengths of various ubiquitous promoters in HEK293T, Cos7 and C2C12 cells. Expression plasmids containing the Fluc coding sequence and different promoters cloned into pcDNA3, which had been previously modified to remove the CMV promoter, were transiently transfected into the different type of cells. An Rluc expression plasmid was co-transfected to control for transfection efficiency and cell number. For comparison, all activities are calculated relative to those obtained from the PGK promoter in the sense orientation. Firefly and *Renilla* luciferase assays were performed one day after transfection. Error bars represent the standard error of the mean from 3 separate experiments.
(TIF)

Figure S2 Confirmation of the activity of the exchange constructs used to generate the different cell lines expressing Fluc under the control of various ubiquitous promoters stably integrated in the *ROSA26* locus. Exchange plasmids were transiently transfected into HEK293T cells along with an Rluc expression construct used to control for transfection efficiency and cell number. For comparison, all activities are calculated relative to those obtained from the PGK promoter in the sense orientation. Firefly and *Renilla* luciferase assays were performed one day after transfection. Error bars represent the standard error of the mean from at least 3 independent experiments.
(TIF)
