# Supplementary figures and images for: A Comparison of Exogenous Promoter Activity at the ROSA26 Locus Using a PhiC31 Integrase Mediated Cassette Exchange Approach in Mouse ES Cells
Source: PLoS One. 2011 Aug 11;6(8):e23376. doi: 10.1371/journal.pone.0023376 (PMC3154917; doi:10.1371/journal.pone.0023376)

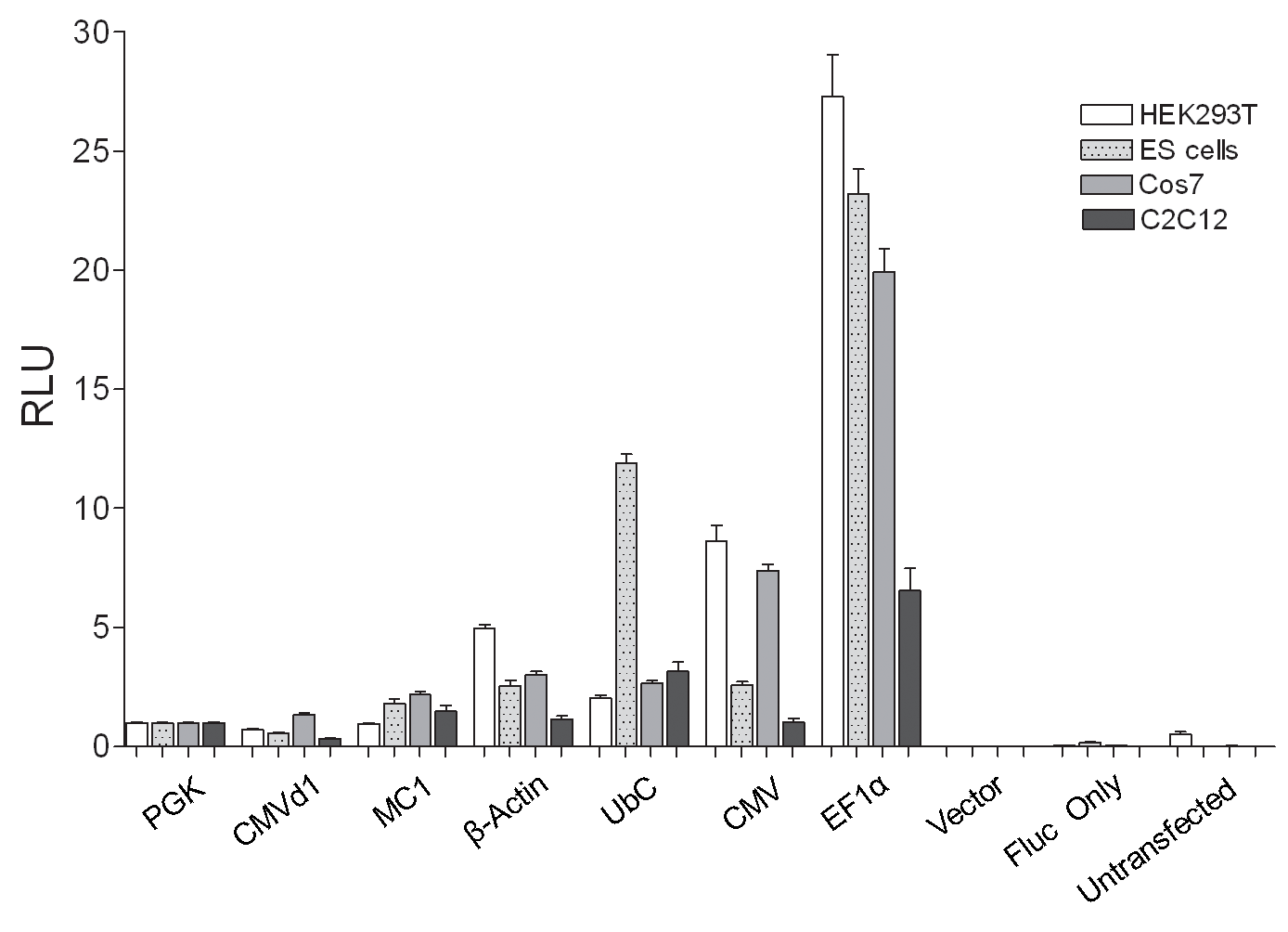

Supplement: Figure S1 — Comparison of the strengths of various ubiquitous promoters in HEK293T, Cos7 and C2C12 cells. Expression plasmids containing the Fluc coding sequence and different promoters cloned into pcDNA3, which had been previously modified to remove the CMV promoter, were transiently transfected into the different type of cells. An Rluc expression plasmid was co-transfected to control for transfection efficiency and cell number. For comparison, all activities are calculated relative to those obtained from the PGK promoter in the sense orientation. Firefly and Renilla luciferase assays were performed one day after transfection. Error bars represent the standard error of the mean from 3 separate experiments. (TIF) [file pone.0023376.s001.tif]

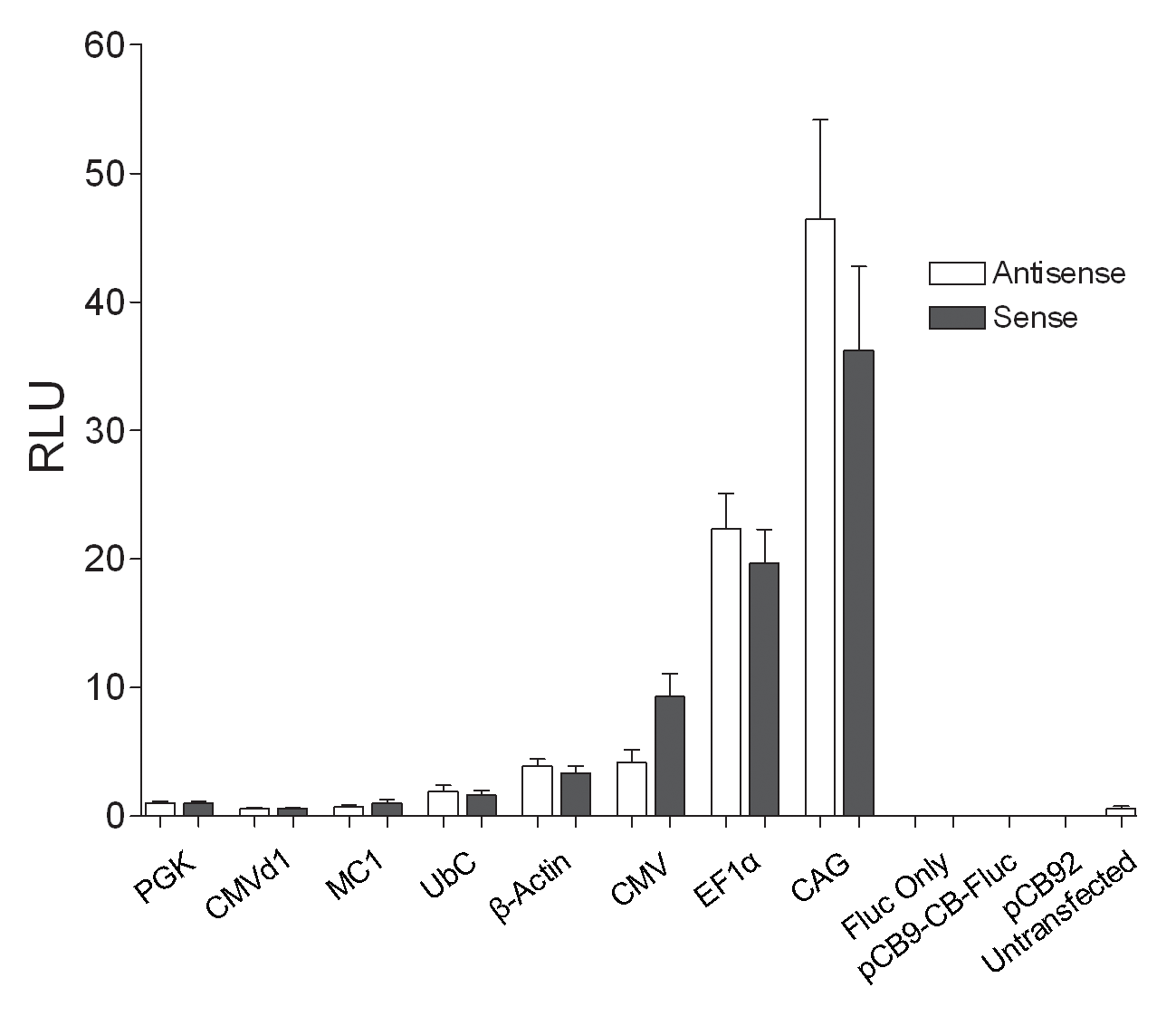

Supplement: Figure S2 — Confirmation of the activity of the exchange constructs used to generate the different cell lines expressing Fluc under the control of various ubiquitous promoters stably integrated in the ROSA26 locus. Exchange plasmids were transiently transfected into HEK293T cells along with an Rluc expression construct used to control for transfection efficiency and cell number. For comparison, all activities are calculated relative to those obtained from the PGK promoter in the sense orientation. Firefly and Renilla luciferase assays were performed one day after transfection. Error bars represent the standard error of the mean from at least 3 independent experiments. (TIF) [file pone.0023376.s002.tif]
